# Investigating the Link between Circadian Clock Gene Expressions, Chronotype, Insomnia, and Daytime Sleepiness in Patients with Obstructive Sleep Apnea

**DOI:** 10.3390/ijms25169062

**Published:** 2024-08-21

**Authors:** Agata Gabryelska, Szymon Turkiewicz, Adrian Gajewski, Piotr Białasiewicz, Dominik Strzelecki, Maciej Chałubiński, Marcin Sochal

**Affiliations:** 1 Department of Sleep Medicine and Metabolic Disorder, Medical University of Lodz, 92-216 Lodz, Poland; turkiewiczszymon99@gmail.com (S.T.); piotr.bialasiewicz@umed.lodz.pl (P.B.); sochalmar@gmail.com (M.S.); 2 Department of Immunology and Allergy, Medical University of Lodz, 92-215 Lodz, Poland; adrian.gajewski@umed.lodz.pl (A.G.); maciej.chalubinski@umed.lodz.pl (M.C.); 3 Department of Affective and Psychotic Disorders, Medical University of Lodz, 92-216 Lodz, Poland; dominik.strzelecki@umed.lodz.pl

**Keywords:** chronotype, OSA, circadian clock, genes, insomnia, sleep medicine

## Abstract

Introduction. This study aimed to investigate the relationship between obstructive sleep apnea (OSA), circadian rhythms, and individual sleep–wake preferences, as measured by chronotype, and to assess the association between circadian clock gene expression and subjective sleep-related variables. Methods: A total of 184 individuals were recruited, underwent polysomnography (PSG), and completed questionnaires including a chronotype questionnaire (CQ), insomnia severity index (ISI), and Epworth sleepiness scale (ESS). Blood samples were collected in the evening before and morning after PSG. Gene expression analysis included BMAL1, CLOCK, PER1, CRY1, NPAS2, and NR1D1. Results: In the OSA group, the subjective amplitude (AM score of CQ) positively correlated with all circadian clock genes in the morning (R ≥ 0.230 and *p* < 0.05 for each one), while the morningness–eveningness (ME score of CQ) was only associated with the evening BMAL1 level (R = 0.192; *p* = 0.044). In healthy controls, insomnia severity correlated with evening expression of BMAL1, PER1, and CRY1. Conclusions: The findings highlight the complex interplay between OSA, circadian rhythms, and sleep-related variables, suggesting potential determinants of morning chronotype in OSA and implicating disrupted circadian clock function in subjective feelings of energy throughout the day. Further research is warranted to elucidate underlying mechanisms and guide personalized management strategies.

## 1. Introduction

The circadian rhythm, governing the 24 h sleep–wake cycle, relies on a central oscillator in the hypothalamus’ suprachiasmatic nuclei. This oscillator synchronizes with the light–dark cycle and oversees peripheral cellular cycles. In humans, the circadian clock comprises two negative feedback loops. The first loop involves activators forming a heterodimer of basic helix-loop-helix ARNT-like protein 1 (BMAL1) and CLOCK/NPAS2 (circadian locomotor output cycles kaput/paralog neuronal PAS domain protein 2), stimulating gene expression, including repressors like periods (PERs) and cryptochromes (CRYs). Their proteins inhibit activators, leading to fluctuating PER and CRY levels, which peak in the evening and are lowest in the morning due to constant degradation. The second loop involves retinoic acid receptor-related orphan receptors (RORs) positively regulating BMAL1 expression, while nuclear receptor subfamily 1 group D member 1 (NR1D1) acts as a transcriptional repressor, influencing RORs and BMAL1. Together, these mechanisms orchestrate the circadian clock, crucial for physiological processes [1].

Disruption of the circadian rhythm can occur due to various factors, including sleep fragmentation, hypoxia, and arousals, which are characteristic of obstructive sleep apnea (OSA) [2] and can manifest as complete abolishment of their daily fluctuations [3,4,5,6], which might affect the chronotype.

OSA is a condition marked by repeated partial or complete blockages of the upper airway during sleep, resulting in disrupted sleep patterns and widespread systemic effects [7,8]. The development of OSA is driven by multiple factors, including anatomical, neuromuscular, and neurobiological elements, which add to the complexity of its management [9,10]. Recent studies have emphasized the potential role of disrupted circadian rhythms and altered neuromodulatory signaling in the underlying mechanisms of OSA [2,11].

Polysomnography (PSG) serves as a crucial diagnostic method for OSA, offering detailed insights into sleep stages, respiratory effort, airflow, and oxygen saturation. This enables the identification of central and obstructive breathing disorders, as well as any expected abnormalities in breathing patterns. The primary metric derived from PSG is the apnea–hypopnea index (AHI), which quantifies the number of apneas and hypopneas per hour of sleep. The AHI is utilized to assess the severity of OSA, categorizing it into mild (5 ≤ AHI < 15), moderate (15 ≤ AHI < 30), and severe (AHI ≥ 30) [12,13].

Chronotype refers to an individual’s preferred timing of sleep and wakefulness, which can range from being an early-morning “lark” to a late-night “owl”. Interestingly, OSA patients are morning- or intermediate-type in the majority [14,15]. Kim et al. showed that morning and evening types were related to OSA severity in older and overweight patients, which suggests that the intermediate chronotype may be a potential protective factor in this specific group [14]. It has been observed that a short sleep duration in actigraphy and less morningness are determinants of a higher risk of sleepiness in newly diagnosed OSA patients [16]. Furthermore, the morning type in OSA is also related to better adherence to continuous positive airway pressure (CPAP) treatment, even after excluding the effect of other factors [17].

To date, there are no known studies investigating the relationship between the circadian clock and chronotype in OSA individuals. However, some indications suggest the existence of an association between both in healthy individuals. For instance, the evening chronotype presents with higher *CLOCK* gene expression compared to the morning type [18]. Moreover, the expression of *PER3*, *BMAL1*, and *CRY1* in healthy individuals differed between the neutral type and morning type in some time points of measurement [19].

Thus, this study aimed to assess the relationship between chosen circadian rhythm-associated questionnaire scores and circadian clock gene expression among OSA patients.

## 2. Results

The baseline demographic, polysomnography, and questionnaire characteristics of the study groups are shown in Table 1. All participants were split into two study groups: control (n = 54; AHI: 1.7 (1.0–3.1)) and OSA (n = 130; AHI: 25.8 (11.7–46.4)).

In the OSA group, the AM score of CQ positively correlated with all circadian clock genes, while the ME score was only associated with the evening *BMAL1* expression level (R = 0.192, *p* = 0.044). All correlations between questionnaire scores and circadian clock gene expressions in the OSA group are presented in Table 2.

In the control group, only the ISI score was associated with the circadian clock gene expressions, specifically evening BMAL1, PER1, and CRY1 (R = 0.339, *p* = 0.015; R = 0.325, *p* = 0.021; and R = 0.345, *p* = 0.019, respectively).

## 3. Discussion

The outcomes of this study shed light on the intricate interplay between OSA, circadian rhythms, and individual sleep–wake preferences, as measured by chronotype. The key finding is the association between all measured circadian clock genes in the morning and the amplitude of chronotype in OSA, but not in healthy controls. No available studies evaluated the relationship between the circadian clock gene expressions and chronotypes in OSA patients. It is known that OSA is characterized by a disruption of circadian clock gene expression and an abolishing of its daily pattern [20,21]. Yang et al. found that especially three genes were disrupted in OSA: *BMAL1*, *CLOCK*, and *CRY2*. Moreover, in the severe form, almost all studied genes’ expressions were downregulated at midnight, including *PER2*, *PER3*, *CLOCK*, *CRY1*, *CRY2*, *BMAL1*, *casein kinase 1ε*, and *timeless* [3]. The possible mechanism of their disruption may be a hypoxia-dependent response [21], mediating via hypoxia-inducible factor (HIF), which was observed in a mouse model [22], as well as in humans [4]. Those changes in circadian clock may alter one’s functioning during the day and lead to intensifying fluctuation in the subjective amplitude of the chronotype. However, it should be mentioned that sleep fragmentation caused by arousals, which are characteristic of OSA [23], may also have an impact on its disruption as well as affect every day functioning, which might be represented through changes in the amplitude of the chronotype [24,25,26].

Additionally, it was observed that the ME score correlated with evening *BMAL1* expression. This means that the morning chronotype may be associated with the evening expression of *BMAL1*. This effect may be caused by the accelerated regeneration of repressor proteins, due to its function [27]. However, it is just a hypothesis that should be confirmed in future studies.

Moreover, the correlation between higher ISI scores and increased evening expression of circadian genes (*BMAL1*, *PER1*, *CRY1*) in the healthy control group is noteworthy. It suggests a potential association between sleep disturbances, particularly insomnia, and circadian dysregulation even in the absence of OSA. This finding aligns with previous research indicating that insomnia may be linked to alterations in circadian rhythms, such as delayed sleep phase and disrupted melatonin secretion [28,29,30].

It is worth mentioning some limitations of this study. First, although the primary circadian clock is located in the suprachiasmatic nucleus of the brain, our study utilized peripheral blood leukocytes as a model for investigating circadian rhythms to study humans with OSA, which has been commonly accepted in the literature. Additionally, circadian clock gene expressions were evaluated only at two time points, which might restrict the potential subtle interplay between chronotype and circadian clock genes. It has to be mentioned that the control and OSA groups differed in demographic data, and this might influence the obtained results; thus, they have to be interpreted with caution. Nevertheless, this was not a case–control study; it involved a center cohort representing a real-life OSA population. Last but not least, the results are limited to the represented population; thus, future studies should involve expansion to particular OSA phenotypes.

## 4. Materials and Methods

A total of 184 individuals were recruited from the Sleep and Respiratory Disorders Centre in Lodz (Poland). The inclusion criteria included informed consent for participation in the study and the PSG examination, age between 18 and 75, and body mass index (BMI) of 20–45 kg/m^2^. The exclusion criteria included lack of consent to participate in the study, inflammatory diseases (e.g., connective tissue diseases or inflammatory bowel diseases), chronic respiratory diseases (e.g., bronchial asthma or chronic obstructive pulmonary disease), any infection within one month of blood collection, diagnosis of cancer (in medical history), diagnosed major neurological conditions, diagnosed psychiatric conditions (e.g., insomnia, narcolepsy), diagnosed sleep disorders other than OSA (e.g., restless leg, non-24 h sleep–wake disorders), caffeine intake >900 mg per day, active smoking or smoking in the past 6 months, and taking medications affecting sleep (e.g., benzodiazepines and melatonin). Participants gave informed consent; this study was approved by the Ethics Committee at the Medical University of Lodz (RNN/432/18/KE).

### 4.1. Polysomnography (PSG)

All participants underwent a physical examination preceding nocturnal PSG recording. PSG (Alice 6, Phillips-Respironics) utilized electroencephalography, electromyography, electro-oculography, thermistor gauge, snoring recordings, body position tracking, piezoelectric gauges, and an electrocardiogram to monitor sleep stages, apnea, hypopnea and arousal events, heart activity, and hemoglobin oxygen saturation (SpO_2_). American Academy of Sleep Medicine guidelines were used to score PSG data. OSA diagnosis and severity were based on the apnea–hypopnea index (AHI).

Blood samples were collected from participants in the evening (15 min before lights out (around 9:00 p.m.) and morning (within 10 min of awakening (around 6:00 a.m.), respectively) using tubes with EDTA, before and after PSG, respectively. The blood samples were stored at −80 °C.

### 4.2. Laboratory Analysis

Each sample underwent RNA extraction from peripheral blood lymphocytes (using TRIzol, Invitrogen, Fisher Scientific Inc., California, CA, USA) and cDNA synthesis (using a dedicated kit according to the protocol provided by a manufacturer (SuperScript IV First-Strand Synthesis System, Thermo Fisher Scientific Inc., California, CA, USA)). The expression levels of the selected genes were measured using qRT-PCR. The reaction mixture included nuclease-free water, TaqMan Fast Advanced Master Mix, cDNA, and gene-specific probes (TaqMan Assays for *BMAL1*, *CLOCK*, *CRY1*, *PER1*, *NPAS2*, *NR1D1*; reference gene: *β-Actin*; Thermo Fisher Scientific Inc., California, CA, USA). CT values were determined, and mRNA expression levels were calculated using 2^−∆∆Ct^ and multiplied by 100.

### 4.3. Questionnaires

In the morning after the PSG examination, the participants completed the following questionnaires.

#### 4.3.1. Chronotype Questionnaire (CQ)

CQ measures circadian rhythm across morningness–eveningness (ME) and subjective amplitude (AM). ME determines if one is more alert in the morning or evening, with lower scores indicating morningness and higher scores indicating eveningness. AM assesses energy level fluctuations, with lower scores indicating minimal diurnal rhythm changes and higher scores indicating significant fluctuations [31].

#### 4.3.2. Insomnia Severity Index (ISI)

The ISI evaluates insomnia severity and its impact on daily life through seven items. It covers sleep onset, maintenance, early awakening, and overall sleep satisfaction. Scores categorize insomnia severity as absent, subthreshold, moderate, or severe [32].

#### 4.3.3. Epworth Sleepiness Scale (ESS)

The ESS evaluates daytime sleepiness and the likelihood of dozing off in various situations. Respondents rate their likelihood of dozing off in eight different scenarios, such as while watching TV or sitting in a car [33].

Statistical analysis was performed with SPSS 28.0 (IBM, Armonk, NY, USA). The level of significance was set at *p* < 0.05. The distribution was evaluated by the Shapiro–Wilk test. All data are presented as median and interquartile range (IQR). Spearman’s rank correlation was used to assess correlations.

## 5. Conclusions

Our study unveils connections between OSA, circadian rhythms, and individual sleep–wake preferences. Circadian clock gene expressions impact energy levels in OSA, which seems to exacerbate chronotype amplitude. Moreover, *BMAL1* expression may be the possible determinant of morning chronotype in OSA. These insights underscore the complexity of sleep disorders and advocate personalized management strategies.

## Figures and Tables

**Table 1:** Baseline demographic, polysomnography, and questionnaire characteristics of study groups. AHI—apnea–hypopnea index; AM—subjective amplitude score of CQ; BMI—body mass index; CQ—chronotype questionnaire; ESS—Epworth sleepiness scale; ISI—insomnia severity index; ME—morningness–eveningness score of CQ; nREM—non-rapid eye movement; REM—rapid eye movement; TST—total sleep time.

Parameter		Control Group (n = 54)	OSA Group (n = 130)	*p*-Value
Demographic data	Age [years old]	44.8 (±12.4)	53.9 (±11.7)	**<0.001**
	Sex (male [n])	36 (66.7%)	108 (83.1%)	**0.005**
	BMI [kg/m^2^]	28.4 (±5.9)	32.2 (±6.0)	**<0.001**
PSG parameters	Sleep efficiency [%]	84.2 (73.6–90.4)	82.7 (74.4–90.0)	0.970
	TST [hours]	6.2 (5.5–7.2)	6.3 (5.4–7.0)	0.813
	REM [hours]	1.3 (0.9–1.7)	1.2 (0.8–1.7)	0.642
	nREM [hours]	4.8 (4.4–5.6)	4.9 (4.3–5.5)	0.990
	Arousal index [events/hour]	9.3 (5.0–12.9)	17.5 (10.9–25.2)	**<0.001**
	AHI [events/hour]	1.7 (1.0–3.1)	25.8 (11.7–46.4)	**<0.001**
	Total number of desaturations	10.5 (6.0–19.8)	146.0 (65.5–298.5)	**<0.001**
	Desaturation index [events/hour]	2.0 (1.0–3.0)	26.0 (11.2–49.5)	**<0.001**
Questionnaires	ESS score	9.0 (5.5–11.0)	8.0 (5.0–12.0)	0.978
	ISI score	14.0 (10.8–18.0)	13.0 (9.0–17.0)	0.199
	ME score of CQ	23.0 (18.0–27.0)	20.0 (17.0–23.0)	**0.002**
	AM score of CQ	23.0 (18.0–26.0)	21.0 (18.0–25.0)	0.216

**Table 2:** Correlations between questionnaire scores and circadian clock gene expressions in the OSA group.

		ME Score of CQ	AM Score of CQ	ISI Score	ESS Score
evening	*BMAL1*	**R = 0.192** ***p* = 0.044**	R = −0.074 *p* = 0.442	R = −0.118 *p* = 0.221	R = 0.069 *p* = 0.475
morning		R = 0.062 *p* = 0.524	**R = 0.257** ***p* = 0.008**	R = 0.130 *p* = 0.183	R = 0.002 *p* = 0.985
evening	*CLOCK*	R = −0.043 *p* = 0.661	R = 0.045 *p* = 0.650	R = −0.106 *p* = 0.277	R = 0.077 *p* = 0.435
morning		R = 0.037 *p* = 0.710	**R = 0.245** ***p* = 0.013**	R = 0.142 *p* = 0.155	R = −0.010 *p* = 0.924
evening	*CRY1*	R = 0.052 *p* = 0.593	R = 0.020 *p* = 0.834	R = −0.050 *p* = 0.606	R = −0.034 *p* = 0.730
morning		R = 0.084 *p* = 0.398	**R = 0.251** ***p* = 0.011**	R = 0.127 *p* = 0.202	R = 0.029 *p* = 0.768
evening	*PER1*	R = −0.126 *p* = 0.199	R = 0.094 *p* = 0.336	R = −0.060 *p* = 0.538	R = 0.138 *p* = 0.158
morning		R = 0.030 *p* = 0.767	**R = 0.259** ***p* = 0.008**	R = 0.143 *p* = 0.151	R = 0.006 *p* = 0.956
evening	*NPAS2*	R = 0.103 *p* = 0.262	R = 0.004 *p* = 0.967	R = −0.062 *p* = 0.501	R = 0.141 *p* = 0.124
morning		R = 0.052 *p* = 0.618	**R = 0.239** ***p* = 0.019**	R = 0.142 *p* = 0.170	R = 0.005 *p* = 0.960
evening	*NR1D1*	R = −0.115 *p* = 0.230	R = 0.001 *p* = 0.996	R = −0.050 *p* = 0.605	R = −0.073 *p* = 0.449
morning		R = 0.046 *p* = 0.636	**R = 0.230** ***p* = 0.017**	R = 0.146 *p* = 0.132	R = −0.002 *p* = 0.981

AM—subjective amplitude; BMAL1—basic helix-loop-helix ARNT-like protein 1; CLOCK—circadian locomotor output cycles kaput; CRY1—cryptochrome 1; CQ—chronotype questionnaire; ESS—Epworth sleepiness scale; ISI—insomnia severity scale; PER1—period 1; NPAS2—paralog neuronal PAS domain protein 2; NR1D1—nuclear receptor subfamily 1 group D member 1.

## Data Availability

The data that support the findings of this study are available from the corresponding author upon reasonable request.
